# Identification of quantitative trait loci (QTLs) and candidate genes of seed Iron and zinc content in soybean [*Glycine max* (L.) Merr.]

**DOI:** 10.1186/s12864-022-08313-1

**Published:** 2022-02-19

**Authors:** Huan Wang, Jia Jia, Zhandong Cai, Mingming Duan, Ze Jiang, Qiuju Xia, Qibin Ma, Tengxiang Lian, Hai Nian

**Affiliations:** 1 The State Key Laboratory for Conservation and Utilization of Subtropical Agro-bioresources, South China Agricultural University, 510642 Guangzhou, Guangdong People’s Republic of China; 2 Guangdong Laboratory for Lingnan Modern Agriculture, 510642 Guangzhou, Guangdong People’s Republic of China; 3 The Key Laboratory of Plant Molecular Breeding of Guangdong Province, College of Agriculture, South China Agricultural University, 510642 Guangzhou, Guangdong People’s Republic of China; 4 Rice Molecular Breeding Institute, GRANLUX ASSOCIATED GRAINS, 518024 Shenzhen, Guangdong, People’s Republic of China

**Keywords:** Soybean, Iron Content, Zinc content, Biofortification, QTL mapping, WGS

## Abstract

**Background:**

Deciphering the hereditary mechanism of seed iron (Fe) and zinc (Zn) content in soybean is important and sustainable to address the “hidden hunger” that presently affects approximately 2 billion people worldwide. Therefore, in order to detect genomic regions related to soybean seed Fe and Zn content, a recombinant inbred line (RIL) population with 248 lines was assessed in four environments to detect Quantitative Trait Loci (QTLs) related to soybean seed Fe and Zn content.

**Result:**

Wide variation was found in seed Fe and Zn content in four environments, and genotype, environment, and genotype × environment interactions had significant influences on both the seed Fe and Zn content. A positive correlation was observed between seed Fe content and seed Zn content, and broad-sense heritability (*H*^*2*^) of seed Fe and Zn content were 0.73 and 0.75, respectively. In this study, five QTLs for seed Fe content were detected with 4.57 - 32.71% of phenotypic variation explained (PVE) and logarithm of odds (LOD) scores ranging from 3.60 to 33.79. Five QTLs controlling the seed Zn content were detected, and they individually explained 3.35 to 26.48% of the phenotypic variation, with LOD scores ranging from 3.64 to 20.4. Meanwhile, 409,541 high-quality single-nucleotide variants (SNVs) and 85,102 InDels (except intergenic regions) between two bi-parental lines were identified by whole genome resequencing. A total of 12 candidate genes were reported in one major QTL for seed Fe content and two major QTLs for seed Zn content, with the help of RNA-Seq analysis, gene ontology (GO) enrichment, gene annotation, and bi-parental whole genome sequencing (WGS) data.

**Conclusions:**

Limited studies were performed about microelement of soybean, so these results may play an important role in the biofortification of Fe and Zn and accelerate the development of marker-assisted selection (MAS) for breeding soybeans fortified with iron and zinc.

**Supplementary Information:**

The online version contains supplementary material available at 10.1186/s12864-022-08313-1.

## Background

Micronutrients are essential nutrient elements required for human growth and development, such as Fe and Zn, because of their vital roles in a great deal of biochemical functions and key metabolic reactions. Fe and Zn are considered to have important biological relevance and clinical significance in global public health [1]. Micronutrient malnutrition is also generally known as “hidden hunger” and affects more than 2 billion people worldwide [2]. For normal human growth and development, the recommended dietary allowances (RDAs) of Fe and Zn are 10-15 mg/d and 12-15 mg/d, respectively [3]. In the world, the majority of people rely on plant foods, which often contain low key micronutrients and do not meet the needs of RDAs, especially for Fe and Zn content [4]. Fe deficiencies can significantly lead to bad influences on human health, such as iron-deficiency anemia, stunting, and heart failure [5, 6]. Zn deficiencies result in the emergence of poor cognitive development, infaust neuronal development, and disordered immunity [7, 8].

Soybean [*Glycine max* (L.) Merr.] is the most prominent food and oil crop between current supply and demand. The micronutrients from soybean seeds are significant portions of the mineral nutrients for humans. Soybean and its products are vital sources of Fe and Zn intake for humans because they have higher content than rice, wheat, and corn [9]. Fortifying the content of Fe and Zn in soybean seeds is significant for alleviating malnutrition in humans whose daily diets lack Fe and Zn. Furthermore, the enrichment of Fe and Zn content in edible parts of crops by biofortification, such as genetic improvement, is considered a sustainable and cost-effective approach to alleviate “hidden hunger” and associated health issues [10, 11].

Understanding the mechanism for the accumulation of Fe and Zn in seeds is vital to increase the seed Fe and Zn content in crops. There are a variety of valuable studies on the seed Fe and Zn content of crops using association analysis and linkage analysis.

Recent years, genome-wide association studies (GWAS) have played a crucial role in identifying significant association locus about seed Fe or Zn content among some crops, such as rice (*Oryza sativa* L.), pearl millet (*Pennisetum glaucum* R. Br.), Mung bean (*Vigna radiata* L.), chickpea (*Cicer arietinum* L.) and so on [12–15]. In contrast, the potential to identify genetic information about seed Fe and Zn content is not underestimated by virtue of linkage analysis. Performing linkage analysis aimed to dissect genetic variability for seed micronutrients by means of RIL population, was demonstrated to be useful and efficient in rice, wheat (*Triticum spelta* L.), common bean (*Phaseolus vulgaris* L.) and pea (*Pisum sativum* L.) [16–21].

However, there are few studies on QTLs related to seed mineral content in soybean, and only several QTLs controlling seed Fe and Zn content have been reported. King et al. [22] found a QTL related to Fe content with LOD score of 4.3 on chromosome 20 (Chr20) using 916 simple sequence repeat (SSR) markers and 92 F_2:4_ lines. Ramamurthy et al. [23] found two QTLs controlling seed Fe content (largest LOD score of 3.79) and three QTLs controlling seed Zn content (largest LOD score of 4.35) using three RIL populations, and all were independently detected once. and Ning et al. [24] detected two QTLs for seed Fe content and five QTLs for seed Zn content, none of them was mapped twice in single environment and the largest LOD score was 5.55. In summary, due to lack of stable and high efficient QTLs, there is still much work to be done to accelerate the development of MAS for breeding soybean cultivars fortified with Fe and Zn.

Deciphering genetic information on these micronutrient traits is essential for breeding Fe- and Zn-rich cultivars by MAS. Linkage analysis is an effective statistical method to anchor the genomic regions related to a specific trait in a segregating population in different environments [25]. Troublesomely, the environment and genotype × environment interaction effects have an influence on the phenotype of the aimed trait in different environments (locations × years), and the QTL × environment interaction (QEI) could also impact the effect and stability of QTLs [26]. Therefore, investigations of these problems contribute to the detection and utilization of QTLs. In addition, with the development of next-generation sequencing (NGS), it is increasingly affordable to identify single nucleotide polymorphism (SNP) markers, which are used to construct high-density genetic maps for QTL mapping. Currently, high-density genetic maps are widely used for genetic analysis in soybean. Cai et al. [27, 28] identified 15 stable QTLs controlling isoflavone content and found a gene (*GmHAD1*) related to low phosphorus tolerance with the help of a high-density genetic map containing 3469 bin markers in soybean. Jiang et al. [29] constructed a high-density map for fine mapping of powdery mildew resistance genes in soybean. Therefore, in this study, we used an RIL population derived from a cross of Guizao 1 × B13 to identify genetic loci and candidate genes related to seed Fe and Zn content in soybean in four environments by means of QTL mapping and whole genome resequencing approaches. The results can be used to breed Fe- and zinc-rich cultivars with the help of MAS and help reduce the “hidden hunger” caused by Fe and Zn malnutrition worldwide.

## Results

### Evaluation of Phenotypic Variation

**Fig. 1:**
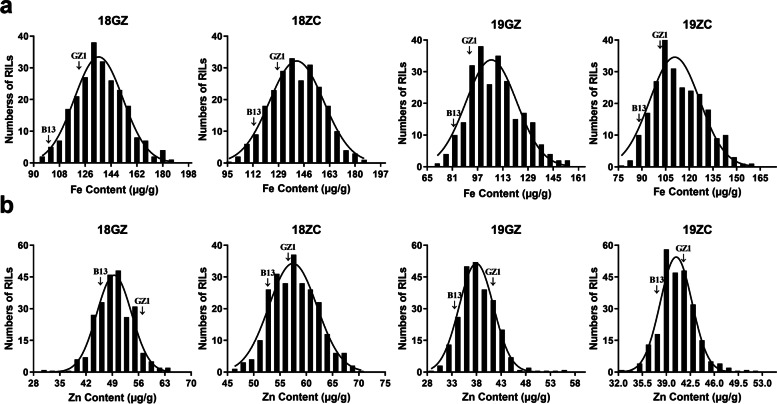
Frequency distribution of Fe and Zn content among the GB RIL population. **a** and **b** indicate the frequency distributions of the Fe and Zn content, respectively. The arrows represent the mean values of the corresponding parents used to construct the GB RIL population (Guizao1 and B13 cultivars)

The parents and GB RIL population exhibited high diversities in seed Fe and Zn content across each environment (18GZ, 18ZC, 19GZ, and 19ZC) (Table 1; Fig. 1S). The ANOVA results demonstrated that widely significant differences existed between GZ1 and B13 for seed Fe content or seed Zn content in the four environments (*p* < 0.05). In the GB RIL population, the variable range of seed Fe content was 72.52 µg g^−1^ to 185.58 µg g^−1^ with a smallest mean of 108.12, largest mean of 136.37 µg g^−1^, largest standard deviation (SD) of 17.08 µg g^−1^, and largest coefficient of variation (CV) of 14.91%. The Zn content varied from 30.01 to 69.50 µg g^−1^, with a smallest mean of 38.32, largest mean of 57.76 µg g^−1^, largest SD of 4.81 µg g^−1^, and largest of CV 9.68%.

**Table 1:** Descriptive statistics of seed Fe and Zn content in parents and GB RIL population

Trait ^*a*^	Envi. ^*b*^	Parents		RIL population								
		GZ1	B13	Mean	SD ^*c*^	Min.	Max.	CV (%)^*d*^	Skew. ^*e*^	Kurt. ^f^	K-S Test	*H* ^*2*^
Fe Content	18GZ	124.47	100.18**	136.37	17.08	98.17	185.58	12.52	0.23	-0.08	0.200*	0.73
	18ZC	130.51	114.09**	141.39	16.54	100.76	184.15	11.70	0.06	-0.48	0.200*	
	19GZ	91.87	83.01**	108.12	16.12	72.52	152.11	14.91	0.42	-0.19	0.023	
	19ZC	104.70	89.95**	113.36	15.39	82.41	159.37	13.58	0.41	-0.43	0.001	
Zn Content	18GZ	58.87	47.01**	49.71	4.81	30.86	63.52	9.68	0.01	0.66	0.200*	0.75
	18ZC	57.65	53.46**	57.76	4.31	47.00	69.50	7.46	0.18	-0.27	0.200*	
	19GZ	42.41	33.75**	38.32	3.54	30.01	55.52	9.24	0.70	1.74	0.200*	
	19ZC	40.86	38.82**	40.64	2.59	32.92	51.30	6.37	0.45	1.16	0.200*	

^a^ The Fe and Zn content is presented as micrograms per gram in the soybean powder. ^b^ Environment (2018 in Guangzhou, 2018 in Zengcheng, 2019 in Guangzhou, and 2019 in Zengcheng are designated as 18GZ, 18ZC, 19GZ, and 19ZC, respectively.). ^c^ Standard deviation. ^d^ Coefficient of variation. ^e^ Skewness. ^f^ Kurtosis

Respecting the normality test, the absolute values of kurtosis and skewness for the two traits across four different environments were all less than two. According to the Kolmogorov-Smirnov test (K-S test) for the two traits in the four environments, the *p* values were all greater than 0.05. Additionally, histograms of the frequency distribution of phenotypic data are depicted in Fig. 1, with wide spans and continuous distributions. All the results above illustrated that the segregation of the seed Fe and Zn content traits fitted normal or skew-normal distribution models, possessing typical quantitative genetic characteristics controlled by multiple genes. Some RILs existed in all environments with lower or higher values than the two parents for seed Fe and Zn content, indicating that transgressive segregation was widely present in the GB RIL population. These results showed that the population was appropriate for QTL mapping because of the large continuous variation.

**Fig. 2:**
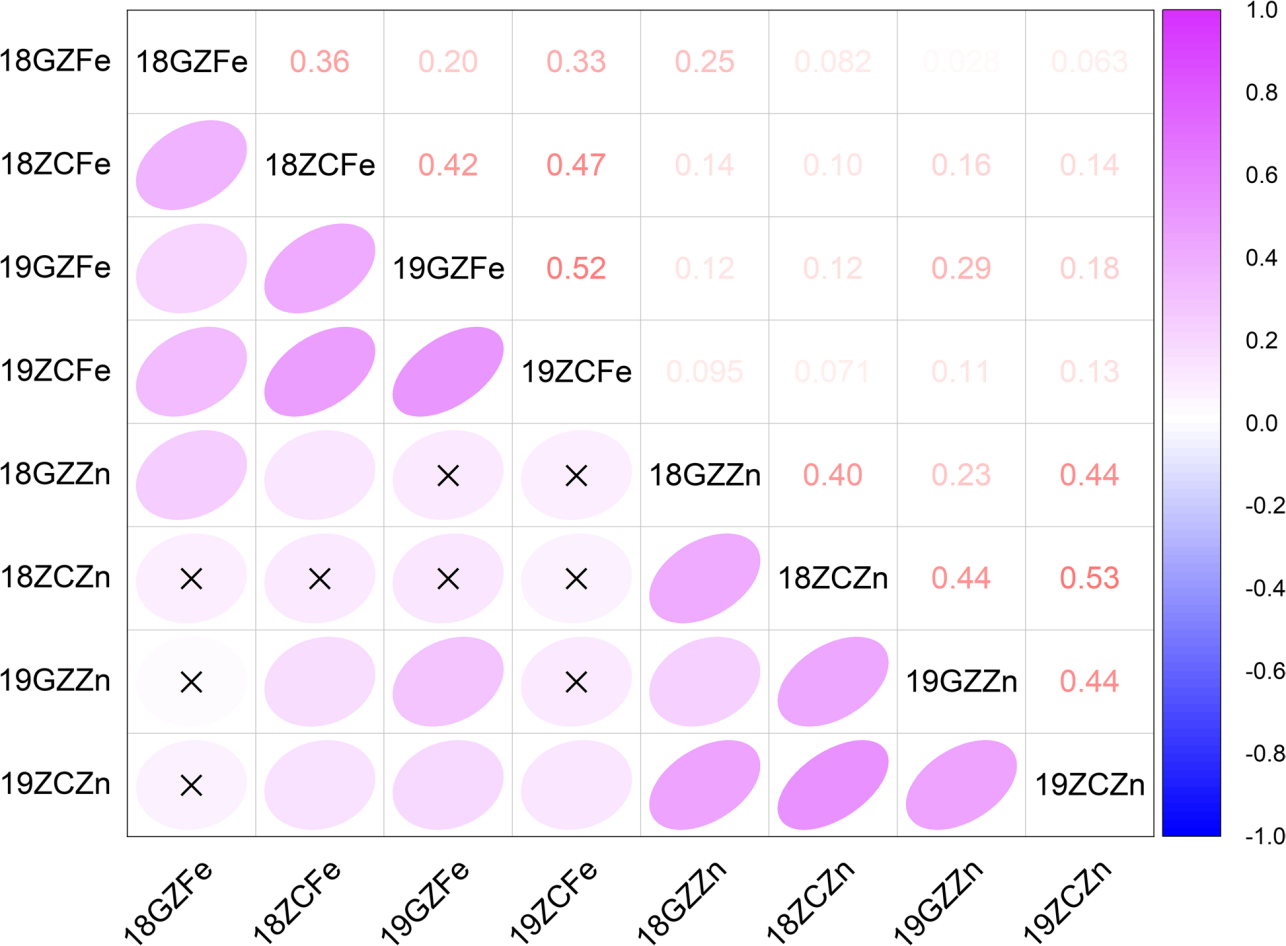
Pearson correlation between the Fe and Zn content of the GB RIL population. Purple and blue indicate positive and negative correlations, respectively. The shape of the cloud and the black cross indicate the correlation strength and no significance, respectively. The values are correlation coefficients. The correlation was significant at the 0.05 level (*p* < 0.05)

The broad-sense heritability (*H*^*2*^) of seed the Fe and Zn content was 73.3% and 75.43%, respectively, in the combined environment (Table 1). High heritability indicated that the genetic variance was superior to other variances, and this population was suitable for high-efficiency selection of high Fe and Zn content cultivars in soybean. The ANOVA results indicated that seed Fe and Zn content were significantly affected by the environment (E), genotype (G), and the interaction between environment and genotype (G × E interaction) (Table 2). ANOVA conducted across the environments showed an extremely significant effect (*P* < 0.01) of G, E, and G × E interactions on both seed Fe and Zn content. The Pearson correlation analysis revealed that seed Fe and Zn content had a significant positive correlation in each environment except 18ZC (Fig. 2). In 18ZC, the nondistinctive and positive correlation between the seed Fe content and seed Zn content could be caused by environmental factors. There was a significant positive correlation of the seed Fe content of the RILs between all the environments, and the relationship of seed Zn content was the same as for the seed Fe content of the RILs.

**Table 2:** Analysis of variance of seed Fe and Zn content across environments

Trait	Source	DF	SS	MS	*F*-value	*P-* value
Fe Content	Block/Environment	4	104864.36	26216.09	131.96	<0.01
	Environment	3	393427.25	131142.42	660.13	<0.01
	Genotype	247	276086.59	1117.76	5.63	<0.01
	G × E interaction	720	241040.22	334.78	1.69	<0.01
Zn Content	Block/Environment	4	11528.76	2882.19	321.44	<0.01
	Environment	3	115052.84	38350.95	4277.13	<0.01
	Genotype	247	16772.77	67.91	7.57	<0.01
	G × E interaction	730	14015.20	19.20	2.14	<0.01

### QTLs Mapping

To guarantee the reliability of detecting QTLs associated with seed Fe and Zn content, CIM, ICIM-ADD, and QEI methods were employed in this study. The significant LOD thresholds of the two traits in single-environment analysis and QEI mapping were determined by 1000 permutations at the level of *p* < 0.05, and the results are listed in Table S1. Through comprehensive analysis of the calculation results of the mapping, the significant LOD thresholds for the two traits were determined to be the same, and 3.6 was determined to be the minimum LOD score for the CIM and ICIM-ADD methods in all environments. In contrast, 5.3 was determined as the minimum LOD score for the QEI method.

#### QTLs Mapping of Seed Fe and Zn Content by CIM method

**Fig. 3:**
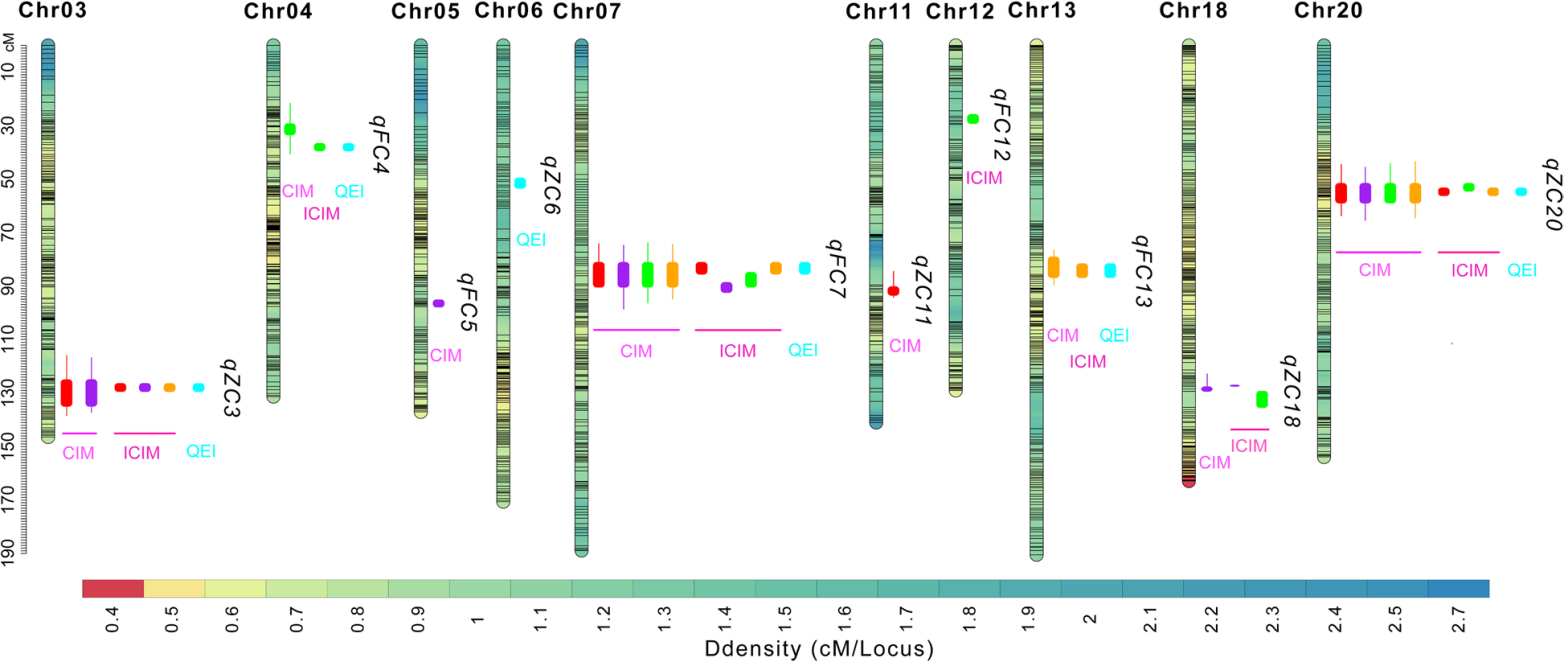
Exhibition of QTL positions on chromosome. Linkage groups are colored and represented according to the marker density. The colors represent a density scale of markers ranging from red (high-density regions) to blue (low-density regions), and the marker position is exhibited by a black line. Ten QTLs for seed Fe and Zn content identified in the GB RIL population are shown on the right side of the corresponding linkage group. Red, purple, green, and orange represent the 18GZ, 18ZC, 19GZ, and 19ZC environments, respectively. Cyan represents the QTLs using QEI mapping analysis

To identify genomic regions associated with the seed Fe and Zn content, a genome-wide scan was performed using the CIM method in Windows QTL Cartographer V2.5. A total of eight QTLs for seed Fe and Zn content were identified in the GB RIL population based on a lingkage genetic map with the phenotypic performances of the four environments (Table 3; Fig. 3).

**Table 3:** Characteristics of QTLs related to seed Fe and Zn content detected using CIM mapping

Trait	Envi. ^*a*^	QTL	Chr ^*b*^	Pos. ^*c*^ (cM)	Marker interval	CI (bp) ^d^	LOD ^*e*^	ADD ^*f*^	PVE (%) ^*g*^
Seed Fe Content	19GZ	*qFC4*	Chr04	28.5	bin35~bin36	5311004~5606879	6.50	-4.82	8.44
	18ZC	*qFC5*	Chr05	96.7	bin108~bin109	35298620~35449207	3.80	3.78	4.90
	18GZ	*qFC7*	Chr07	79.4	bin87~bin91	8446317~9568439	6.40	-5.41	10.00
	18ZC	*qFC7*	Chr07	90.0	bin87~bin91	8446317~9568439	16.30	-8.08	23.76
	19GZ	*qFC7*	Chr07	87.2	bin87~bin91	8446317~9568439	12.40	-6.77	17.48
	19ZC	*qFC7*	Chr07	84.4	bin87~bin91	8446317~9568439	23.60	-8.47	29.86
	19ZC	*qFC13*	Chr13	85.7	bin106~bin108	25905367~26941818	4.90	-3.81	5.09
Seed Zn Content	18GZ	*qZC3*	Chr03	128.5	bin157~bin167	42773578~43911616	8.80	-1.74	12.79
	18ZC	*qZC3*	Chr03	124.4	bin157~bin167	42773578~43911616	7.90	-1.30	8.94
	18GZ	*qZC11*	Chr11	89.6	bin78~bin79	11209422~11357194	4.50	1.45	6.28
	18ZC	*qZC18*	Chr18	124.4	bin205~bin207	51866863~52524554	4.60	0.99	5.04
	18GZ	*qZC20*	Chr20	55.0	bin56~bin75	13374481~33919927	6.70	1.50	9.57
	18ZC	*qZC20*	Chr20	56.3	bin56~bin75	13374481~33919927	20.40	2.24	26.48
	19GZ	*qZC20*	Chr20	53.4	bin56~bin75	13374481~33919927	4.40	0.96	6.98
	19ZC	*qZC20*	Chr20	55.0	bin56~bin75	13374481~33919927	15.80	1.21	21.43

^a^ Environment; ^b^ Chromosome; ^c^ Position of the peak of the QTL in chromosomes; ^d^ Confidence interval; ^e^ Logarithm of odds; ^f^ Additive effect of QTL; ^g^ Percent of phenotypic variance explained

For the seed Fe content, four QTLs, *qFC4*, *qFC5*, *qFC7*, and *qFC13*, were detected on Chr04, Chr05, Chr07, and Chr13, respectively. Their LOD scores ranged from 3.8 to 23.6, with phenotypic variation explained (PVE) ranging from 4.90 to 29.86%. *qFC5* with positive additive effects (ADDs) acquired the favorable allele from the female parent Guizao1. *qFC4*, *qFC7*, and *qFC13* showed negative ADDs and acquired the favorable allele from the male parent B13. Of these QTLs, *qFC4*, *qFC5*, and *qFC13* were only identified in 19GZ, 18ZC, and 19ZC, respectively, while *qFC7* could be detected in all environments and the PVE reached its highest value of 29.86% in 19ZC.

Four QTLs (*qZC3*, *qZC11*, *qZC18*, and *qZC20*) controlling the seed Zn content were detected on Chr03, Chr11, Chr18, and Chr20. They individually explained 5.04 to 26.48% of the phenotypic variation, with LOD scores ranging from 4.4 to 20.4. *qZC11* and *qZC18* were detected in 18GZ and 18ZC, respectively. *qZC3* could be detected in 18GZ and 18ZC, while *qZC20* could be detected in all environments. *qZC3* had the largest PVE of 12.79% in 18GZ, and *qZC20* had the largest PVE of 26.48% in 18ZC. *qZC3* had a negative ADD with a favorable allele from the male parent B13, and the favorable alleles of the remaining three QTLs were contributed by Guizao1.

#### QTLs Mapping of Seed Fe and Zn Content by ICIM-ADD method

The ICIM method in the IciMapping program V4.2 was also concurrently employed in this study. Four QTLs for seed Fe content and three QTLs for seed Zn content were identified (Table 4; Fig. 3). Four QTLs (*qFC4*, *qFC7*, *qFC12*, *and qFC13*) for seed Fe content were located on Chr04, Chr07, Chr12, and Chr13, respectively. The LOD scores ranged from 3.6 to 26.83, and the PVE ranged from 4.57 to 32.41%. The ADD of those QTLs was negative, showing that the favorable alleles were contributed by B13. *qFC4*, *qFC12*, and *qFC13* were identified in 19GZ, 19GZ, and 19ZC, respectively. *qFC7* could be detected in all environments, with the largest PVE at 32.41% in 19ZC.

**Table 4:** Characteristics of QTLs related to seed Fe and Zn content detected using ICIM mapping

Trait	Envi. ^*a*^	QTL	Chr. ^*b*^	Pos. ^*c*^ (cM)	Marker interval	CI (bp) ^d^	LOD ^*e*^	ADD ^*f*^	PVE (%) ^*g*^
Fe Content	19GZ	*qFC4*	Chr04	38.2	bin46~bin47	6194153~6689660	6.13	-4.69	8.00
	18GZ	*qFC7*	Chr07	84.0	bin87~bin88	8446317~8563902	6.92	-6.08	12.61
	18ZC	*qFC7*	Chr07	90.0	bin92~bin93	9568440~9745745	15.74	-8.28	25.33
	19GZ	*qFC7*	Chr07	87.0	bin90~bin91	8633014~9568439	12.69	-7.05	18.19
	19ZC	*qFC7*	Chr07	84.1	bin87~bin88	8446317~8563902	26.83	-9.01	32.41
	19GZ	*qFC12*	Chr12	28.0	bin27~bin28	4498589~4568321	3.60	-3.53	4.57
	19ZC	*qFC13*	Chr13	85.2	bin107~bin108	26336429~26941818	6.07	-3.94	6.19
Zn Content	18GZ	*qZC3*	Chr03	128.2	bin158~bin159	42959977~43331949	7.75	-1.69	11.69
	18ZC	*qZC3*	Chr03	128.2	bin158~bin159	42959977~43331949	7.29	-1.31	8.48
	19ZC	*qZC3*	Chr03	128.2	bin158~bin159	42959977~43331949	3.73	-0.56	4.98
	18ZC	*qZC18*	Chr18	127.0	bin204~bin205	51698565~51939931	3.78	0.93	4.23
	19GZ	*qZC18*	Chr18	135.0	bin207~bin208	52009904~52593803	3.64	0.87	6.84
	18GZ	*qZC20*	Chr20	55.0	bin62~bin63	25069476~27890103	5.98	1.47	8.72
	19GZ	*qZC20*	Chr20	53.1	bin56~bin57	13374481~22252230	3.82	0.90	7.35
	19ZC	*qZC20*	Chr20	55.0	bin62~bin63	25069476~27890103	15.14	1.20	22.28

^a^ Environment; ^b^ Chromosome; ^c^ Position of the peak of the QTL in chromosome; ^d^ Confidence interval; ^e^ Logarithm of odds; ^f^ Additive effect of QTL; ^g^ Percent of phenotypic variance explained

Three QTLs (*qZC3*, *qZC18*, and *qZC20*) controlling the seed Zn content were identified on Chr03, Chr18, and Chr20. They individually explained 4.23 to 22.28% of the phenotypic variation with LOD scores ranging from 3.64 to 15.14. Surprisingly, those QTLs could be identified in multiple environments. *qZC18* was detected in 18ZC and19GZ; *qZC3* could be identified in 18GZ, 18ZC, and 19ZC; and *qZC20* could be identified in 18GZ, 19GZ, and 19ZC. *qZC3*, *qZC18*, and *qZC20* had the largest PVEs of 11.69%, 6.84%, and 22.28%, respectively. *qZC3* had a negative ADD with a favorable allele from the male parent B13, and the favorable alleles of *qZC18* and *qZC20* were contributed by Guizao1.

#### QEI mapping

Under the MET functionality of the IciMapping program V4.2, three QTLs related to seed Fe and Zn content were identified by the multi-environment QTL analysis method (Table 5; Fig. 3). Three QTLs (*qFC4*, *qFC13*, and *qFC7*) for the seed Fe content present on Chr04, Chr07, and Chr13 had LOD scores ranging from 6.68 to 33.79, and the total PVE ranged from 4.80 to 32.71%. *qFC4* and *qFC13* displayed high phenotypic variation contributed by additive × environment effects (PVE (A × E)), which was demonstrated by the high LOD score from the QTL × environment interaction (LOD (A × E)) and the low LOD score from the additive effect (LOD (A)). In contrast, the phenotypic variation contributed by additive effects (PVE (A)) of *qFC7* was more powerful than PVE (A × E), showing its great stability across environments.

**Table 5:** Characteristics of QTLs related to seed Fe and Zn content detected using QEI mapping

Trait	QTL	Chr. ^*a*^	Pos. (cM) ^*b*^	Marker interval	LOD ^*c*^	LOD (A) ^*d*^	LOD (A × E) ^*e*^	PVE ^*f*^	PVE (A) ^*g*^	PVE (A × E) ^*h*^	ADD ^*i*^	QTL × environment interaction			
												A × E1 ^*j*^	A × E2 ^*k*^	A × E3 ^*l*^	A × E4 ^*m*^
Fe Content	*qFC4*	Chr04	38.2	bin46~bin47	7.47	0.53	6.94	7.86	0.53	7.33	-0.67	2.54	1.63	-4.00	-0.17
	*qFC7*	Chr07	84.0	bin87~bin88	33.79	16.46	17.33	32.71	16.94	15.77	-3.80	-1.99	3.20	3.80	-5.02
	*qFC13*	Chr13	85.2	bin107~bin108	6.68	2.38	4.30	4.80	2.32	2.48	-1.41	1.52	0.71	0.14	-2.37
Zn Content	*qZC3*	Chr03	128.2	bin158~bin159	20.88	19.49	1.39	17.98	14.82	3.16	-1.01	-0.62	-0.27	0.45	0.45
	*qZC6*	Chr06	51.0	bin43~bin44	5.47	3.55	1.92	3.35	2.58	0.77	0.42	-0.26	-0.04	0.37	-0.07
	*qZC20*	Chr20	55.0	bin62~bin63	20.96	8.26	12.71	12.54	6.14	6.40	0.65	0.77	-0.66	-0.66	0.55

^a^ Chromosome; ^b^ Position of the peak of the QTL in chromosome; ^c^ Overall LOD score of the QTL; ^d^ LOD score from additive effect; ^e^ LOD score from QTL × environment interaction; ^f^ Percent of phenotypic variance explained; ^g^ Percent of phenotypic variance explained by additive effect, ^h^ Percent of phenotypic variance explained by QTL × environment interaction; ^i^ Average additive effect of QTL; ^j^ QEI effect in E1 (18GZ); ^k^ QEI effect in E2 (18ZC); ^l^ QEI effect in E3 (19GZ); ^m^ QEI effect in E4 (19ZC)

Three QTLs (*qZC3*, *qZC6*, and *qZC20*) controlling the seed Zn content were identified on Chr03, Chr06, and Chr20. They individually explained 3.35 to 17.98% of the total phenotypic variation, and the total LOD scores ranged from 5.47 to 20.96. *qZC3* and *qZC6* were equipped with stronger PVE (A) than PVE (A × E), as indicated by the high LOD (A) compared to the low LOD (A × E), which shows their excellent stability across environments. *qZC20* exhibited a PVE (A) almost equal to PVE (A × E).

In combination with the results of the CIM and ICIM-ADD mapping, *qFC7* with largest PVE of 32.41% and *qZC20* with largest PVE of 26.48% could be repeatedly detected at least three times in the CIM and ICIM-ADD methods, and they could be detected in the MET analysis with high genetic effects. Although *qZC20* exhibited a slightly smaller PVE (A) than PVE (A × E), it could be detected three and four times in the CIM and ICIM-ADD maps, respectively. *qZC3* with largest PVE of 12.79% was detected three times and two times in the ICIM-ADD and CIM methods, respectively. Additionally, *qZC3* with stronger PVE (A) was mapped in the MET analysis, *qZC3* was a major QTL. Therefore, we will mainly focus on these three major QTLs in the follow-up analysis. From results of three mapping methods, among *qFC7*, *qZC3*, and *qZC20*, every QTL from single environment was overlapping on either one corresponding locus, and peaks of any two QTL from single environment distanced less than 5 cM for *qZC3* and *qZC20*. One or two peaks of QTL slightly distanced more than 5 cM, but they were mostly overlapping. Interestingly, all confidence interval (CI) of QTLs from ICIM-ADD and MET methods were included in corresponding QTL CI from CIM method. Hence, the results of CIM method were mainly used as basic of identifying candidate genes. Although there were larger QTL CIs, it was worthy for high reliability of further analysis.

#### Whole genome resequencing of two parents

To identify accurate candidate genes, the parental lines were resequenced at the whole-genome scale. GC content of Guizao1 was 37.5%, which of B13 was 37.3%. 92.5 and 115.9 million high-quality reads were obtained from Guizao1 and B13, respectively. 98.89% of reads were mapped to reference genome Wm82.a2.v1 (http://www.soybase.org/) for Guizao1, and it was 98.34% for B13. The average depths of Guizao1 and B13 were respectively 9.0× and 11.27×. Based on analysis, there were 409,541 high-quality single-nucleotide variants (SNVs) (except intergenic regions) between two bi-parental lines, and nonsynonymous SNVs were 29,548. Similarly, 85,102 InDels were identified and 2490 of which were in coding sequence.

#### Candidate genes prediction of the major QTLs

The release of the soybean reference genome Wm82.a2.v1, whole-genome resequencing, and gene annotations made it efficient to recognize candidate genes in the genetic regions related to the two traits. In this study, three stable and major QTLs; *qFC7*, which was related to seed Fe content; and *qZC3* and *qZC20*, which were related to seed Zn content, were deemed the most promising genomic regions. *qFC7*, *qZC3*, and *qZC20* harbored 108, 151, and 403 genes, respectively, and these all-model genes were downloaded from Phytozome and SoyBase (Table S2). The OmicShare Tools was used to perform GO enrichment analysis, which showed that the main enriched GO terms included cellular process, single-organism process, metabolic process, biological regulation, developmental process, multicellular organismal process, regulation of biological process, cell, signaling, cell part, organelle, membrane, catalytic activity, binding, transporter activity and so on (Table S3).

To reduce the number of candidate genes and identify candidate genes, based on the resequencing analysis of Guizao1 and B13, a total of 41,318 different variants (38,151 SNVs and 3,167 InDels) could be found in three QTL regions concerning Guizao1 and B13 (Tables S4 and S5). Among them, 475 SNVs and 15 InDels were in coding sequence. Further analysis showed that 44, 41, and 125 putative functional mutant genes were obtained in the regions of *qFC7*, *qZC3*, and *qZC20*, respectively.

To further refine these putative candidate genes, the freely available RNA-Seq data of 210 mutant genes from SoyBase were analyzed (Table S6, Fig. 2S) to exclude non-expressed or very low expressed genes. According to the analysis above, in combination with the GO enrichment analysis, different variants, RNA-Seq data, gene annotations, and available literature, three of 41 genes in the *qFC7* genetic region (*Glyma.07G093700*, *Glyma.07G097700*, and *Glyma.07G100700*) were predicted as candidate genes associated with seed Fe content in soybean, while three of 44 genes in the *qZC3* block (*Glyma.03G229400*, *Glyma.03G231200*, and *Glyma.03G240000*), and six of 125 genes in the *qZC20* genetic region (*Glyma.20G063100*, *Glyma.20G068300*, *Glyma.20G067300*, *Glyma.20G076700*, *Glyma.20G088600*, and *Glyma.20G089300*) were predicted as candidate genes related to seed Zn content in soybean (Table S7).

## Discussion

Micronutrient deficiencies, particularly the lack of Fe and Zn, which influence billions of people’s health worldwide, are termed “hidden hunger”. This is a serious health problem for humans, especially in developing and low-income countries [2]. Effective measures, such as biofortification, need to be taken to obtain rich mineral foods to solve micronutrient malnutrition or hidden hunger due to its promising and cost-efficient characteristics [10, 11]. A variety of teams have interest in pursuing increased seed mineral content of crops such as maize, wheat, rice, and beans [30]. QTL mapping is helpful to dissect the genetic architecture of seed micronutrient content by finding markers or genes associated with seed mineral content in crops. In the current study, we detected one major QTL and three potential candidate genes for seed Fe content and two major QTLs for seed Zn content with nine potential candidate genes. Understanding the genetic mechanism of increasing the seed Fe and Zn content is a prerequisite to breed mineral-rich cultivars using MAS in soybean.

### Variations of seed Fe and Zn content in soybean

Genetic variation has a great influence on QTL mapping. A number of studies on mineral variation have been reported in many crops, such as wheat, rice, common bean, and soybean. Our wide variation of seed Fe and Zn content corroborated those findings [16–19, 23, 24]. Our study also showed that genotype, environment, and genotype × environment interactions had significant influences on both the seed Fe and Zn content (Table 3), which was similar to previous studies [15, 31, 32]. Among them, the influences of environmental and genotype × environment interactions increased the difficulty of identifying real QTLs of the desired traits because the phenotype could be inconsistent with the true genotype. In this study, the *H*^*2*^ values of seed Fe and Zn content were high, that was moderate to high compared with a previous study in soybean [24]. In other crops, there are similar results for *H*^*2*^ of seed Fe and Zn content [31, 33]. High heritability means that the phenotype is to a great extent shaped by the genotypic effect.

Correlation analysis among quantitative traits is necessary to estimate the feasibility that multiple traits are jointly selected in breeding. Plant breeders can efficiently improve both traits or multiple traits simultaneously with the help of positive genetic correlation between two or more desirable traits [34]. Understanding the genetic correlation between seed Fe and Zn content can assist plant breeders in designing an appropriate breeding approach to increase both seed Fe and Zn content in soybean at the same time. In this study, correlation between seed Fe content seed Zn content was similar to previous studies [22–24]. We can infer that the accumulation of Fe and Zn in soybean seeds might share the same molecular mechanisms, even in other crops. Similar relationships occurred in rice, wheat, common bean, and spinach [17–19, 35, 36]. which indicates that it is realistic to simultaneously improve different mineral content in crop breeding.

### QTL mapping and comparison with previous studies

A previous study explained that the most suitable mapping procedures were different for the presence of different genetic model data in practical experiments [37]. In recent years, as a variety of QTL mapping methods and software have been invented and improved, an increasing number of algorithms for mapping QTLs have been employed. The mapped QTL accuracy is easily influenced by the mapping procedures, and an unsuitable method could result in the appearance of erroneous or false-positive results [38]. A QTL that can be simultaneously detected by at least two different mapping methods might have high reliability [37].

CIM, ICIM-ADD, and QEI procedures were employed to ensure the reliability of the detected QTLs. Moreover, both traits had significant differences between the two parents that were distant geographical species in the same environment, and the RILs were repeatedly planted in the same location and in different years. This allowed us to identify the environmental stability of the QTLs. Similarly, crop breeders usually identify genetic materials with the desired traits in various environments again to eliminate environmental effects and verify stable genotypes. Several researchers have carried out QTL mapping in multiple environments with locations × years by using at least two mapping methods and obtained good results [38, 39].

Non-simultaneous identification of QTLs among procedures or environments demonstrates that there may be the differential expression of genes that are environmentally specific, which results in QTL × environment interactions [26]. For seed Fe content, it should be noted that four minor QTLs (*qFC4*, *qFC5*, *qFC12*, and *qFC13*) were only detected in one environment or by one method in single-environment analysis. For the former, those QTLs could be unstable and environment-specific; for the latter, those different QTLs were identified and could be caused by the different detection power of mapping procedures [37]. Although *qFC4* and *qFC13* could be detected in QEI mapping analysis, they possessed a higher PVE (A × E) than PVE (A), and they were susceptible to the environment and unstable. For the seed Zn content, *qZC6* had a higher PVE (A) than PVE (A × E), and in single-environment analysis, small peaks with LOD scores lower than the threshold of 3.6 could be found adjacent to this locus in several environments. it is possible that *qZC6* had a minor effect and was not identified in the single-environment analysis but was identified in the QEI; such a case was similar to a previous study [40].

A few QTLs related to soybean seed Fe and Zn content have been detected by several researchers in recent years [22–24]. A stable QTL was detected on Chr3 for seed Fe content and a stable QTL was detected on Chr11 for seed Zn content; other QTLs were only detected in the special environment and not repeated in another study. In the current study, three major QTLs (*qFC7* for seed Fe content and *qZC3* and *qZC20* for seed Zn content) were new loci, and other QTLs were not similar to previous results. This may be due to the following reasons: (i) previous studies detected few relevant QTLs on account of the lack of high-quality linkage maps; (ii) there are immense genetic differences between mapping populations; and (iii) QTL mapping is influenced by environmental effects, population size, and experimental error.

### Putative candidate genes of interest in three major QTLs

In previous studies, some basic helix-loop-helix (bHLH) transcription factors, myeloblastosis (MYB) transcription factors, zinc transporter proteins (ZIPs), nicotianamine synthases (NASs), YS-like (YSL) transporters, oligopeptide transporters (OPTs), RING zinc finger protein, and hemerythrin motif-containing RING- and zinc-finger (HRZ) proteins were demonstrated to be involved in the uptake, transport, and storage of Fe and Zn in plants [41]. Nonsynonymous sequence variations could change protein sequence, which possibly effect gene or protein function. The identify of SNV plays an importance role in genome-assisted research and QTL mapping [42–44].

Nevertheless, *Glyma.03G231200*, *Glyma.20G063100*, *Glyma.20G068300*, and *Glyma.20G088600* do not have nonsynonymous SNV or InDel mutations that can cause changes in proteins. Interestingly, their homologous genes, *OsNAS2*, *OsZIP9*, *YSL1*, and *bHLH104*, play important roles in Fe and Zn homeostasis in other crops. Zn and Fe concentrations of unpolished rice were significantly increased in *OsNAS2* overexpression populations compared with controls [45]. Yang et al. [46] demonstrated that *OsZIP9* was responsible for transporting Zn from external media into root cells, and *GmZIP1* could facilitate the uptake of Zn in yeast cells [47]. Zn, Cu, and Fe were aberrantly distributed in Arabidopsis when *YSL1* and *YSL3* were nonfunctional [48]. *bHLH104* and *bHLH34* were demonstrated to positively regulate Fe homeostasis in *Arabidopsis thaliana* [49]. Most likely, there are some complex structural variations, epigenetic modifications, and enhancers resulting in their differential expression between parents, so *Glyma.03G231200*, *Glyma.20G063100*, *Glyma.20G068300*, and *Glyma.20G088600* could be related to the seed Zn content in soybean. *Glyma.03G240000* is a bHLH transcription factor and *Glyma.03G229400* encodes glucose-6-phosphate 1-dehydrogenase 4, which might participate in Zn homeostasis in soybean. *Glyma.07G100700* and *Glyma.07G097700* is a MYB transcription factor and RING zinc finger protein, respectively. *Glyma.07G093700* encode HRZ proteins, and *OsHRZ1*, the homologous gene of *Glyma.07G093700*, is a putative iron-binding sensor that regulates rice responses to Fe deficiency [50]. Therefore, these genes might be responsible for increasing the seed Fe content in soybean. *Glyma.20G076700* and *Glyma.20G067300* encode RING zinc finger protein and OPT proteins, respectively. The annotation of *Glyma.20G089300* is responsible for metal ion binding. So, these three candidate genes might play important roles in increasing seed Zn content in soybean. To sum up, all 12 candidate genes may be important genetic resources of improving Fe- or Zn- rich soybean cultivars, however they should be further validated.

## Conclusions

In conclusion, we have used an RIL population derived from a cross of Guizao 1 × B13 to identify QTLs and candidate genes related to seed Fe and Zn content in soybean in four environments by means of three QTL mapping approaches and whole-genome resequencing data of two parents. these QTLs and candidate genes related to seed Fe and Zn content can provide vital genetic information to breed Fe- and Zn-fortified cultivars with the help of MAS in soybean. Promisingly, Fe and Zn biofortification projects are being conducted, and several candidate genes are in the process of functional validation to ascertain their effects for increasing seed Fe and Zn content in soybean.

## Methods

### Plant Materials and Field Experiments

The mapping population (GB RIL population) was developed by crossing Guizao 1 and BRSMG68 (‘B13’ hereafter) and consisted of 248 lines. Guizao1 is a cultivar bred by the Cash Crops Research Institute, Guangxi Academy of Agricultural Sciences, which is rich in mineral elements. B13 was introduced from Brazil with a low content of mineral elements. Both parents and GB RILs were provided by the Guangdong Subcenter of the National Center for Soybean Improvement and South China Agricultural University, Guangzhou, China.

The F_8:11−12_ generations of GB RILs together with the two parents were planted at two the following different locations in July 2018 and in July 2019: Guangzhou Experimental Station (N23° 15′, E113° 34′) in Guangdong and Zengcheng Experimental Station (N23° 24′, E113° 64′) in Guangdong. The four environments (2018 at Guangzhou Experimental Station, 2019 at Guangzhou Experimental Station, 2018 at Zengcheng Experimental Station, and 2019 at Zengcheng Experimental Station) were assigned as 18GZ, 19GZ, 18ZC, and 19ZC, respectively. All GB RILs and their two parents were planted in a single-line plot of 2 m in length, 0.5 m in width, and 0.1 m between plants in a randomized complete block design with two replicates. In each environment, field management followed normal soybean production practices for the area. In the end, this study absolutely complies with relevant institutional, national, and international guidelines.

### Determination and Analysis of Phenotypic Data

The seed Fe and Zn content in soybean were determined by atomic absorption spectrometry (AAS, Model AA-6800, Shimadzu, Japan) using the wet digestion method. In brief, approximately 100 whole seeds that were randomly selected from the full plot representing each line were ground into a fine powder by a grinder, and then each sample was overdried at 40 °C for approximately 72 h to a constant mass. Then, 0.5 g of dried soybean powder was coldly digested with 10 ml of a diacidic mixture of HNO_3_:HClO_4_ (4:1 v/v) for 12 h, followed by heat treatment. The next digestion procedure was set as follows: raising from room temperature to 120 °C and holding at 120 °C for one hour; raising from 120 to 180 °C and holding at 180 °C for 30 min; and then finally raising from 180 to 240 °C and holding at 240 °C to thorough digestion. The resulting solutions were diluted to 50 ml with ultrapure water and then filtered after cooling to room temperature. The Fe and Zn content were determined using AAS.

All phenotypic data were analyzed. Descriptive statistics and Pearson correlations were performed by the Origin21 software (OriginLab Corporation, Northampton, USA.). Frequency distribution graphs of seed Fe and Zn content were depicted by GraphPad Prism 9.0 (GraphPad Software, Inc., USA.). The broad-sense heritability (*H*^*2*^) estimation was calculated using the following equation:

$${H}^2={\sigma}_G^2/\left({\sigma}_G^2+{\sigma}_{GE}^2/E+{\sigma}_e^2/ rE\right)\times 100\%$$

where σ^2^_G_ is the genotypic variance, σ^2^_GE_ is the genotype × environment interaction variance, σ^2^_e_ is the error variance, *E* is the number of environments, and r is the number of replicates within an environment.

### QTL Detection

The genetic map employed in the current study was constructed in our previous study, including 3748 bins and 3031.9 cM in length, with an average distance of 0.81 cM between adjacent markers on 20 chromosomes [29]. QTL analysis was carried out by composite interval mapping (CIM) in Windows QTL Cartographer V2.5 and inclusive composite interval mapping (ICIM-ADD) in IciMapping V4.2 [51, 52]. QTL × environment interaction (QEI) mapping for multienvironmental trials was performed using ICIM-ADD [40]. The LOD thresholds of significant QTLs were determined by performing 1000 permutations using a Type I error set at *p* < 0.05. QTL mapping results were comprehensively compared to SoyBase (http://www.soybase.org/). The physical locations of QTLs were drawn on related chromosomes using the LinkageMapView package in R [53]. Standard nomenclature was involved in naming QTLs [54]. QTL detected at least three times by either one of CIM and ICIM-ADD methods was termed as a stable QTL. QTL that was detected with PVE of more 10% in CIM or ICIM-ADD methods and could be simultaneously detected in MET method, which was categorized a major QTL [55, 56]. In this study, QTLs with large shared position or peaks of adjacent QTL distanced to less than 5 cM were regarded as the same QTL [38].

### Whole genome resequencing of two parents

WGS approach was implemented in bi-parental genome using Illumina HiSeq2000 at the Beijing Genome Institute (BGI) Tech, Shenzhen, China [57], details of technique and analysis are as described in Jiang et al. [29]. In brief, using the method of CTAB extracted the genomic DNA of two parent [58]. Illumina libraries were built according to Illumina protocol, and 90-bp paired-end reads were produced. Using Burrows-Wheeler Alignment tool (BWA), sequenced reads were mapped the Wm82. a2. v1 (soybean reference genome) from SoyBase (http://www.soybase.org/) [59, 60]. Aligned reads were dealt with samtools, and Picard software was used to remove PCR duplicates of aligned reads. Finally, high-confidence SNVs and InDels were obtained by using (GATK) [61].

### Candidate Genes Identification

In the current study, the gene models that fell into mapping intervals of detected QTLs were retrieved from Wm82.a2.v1 (soybean reference genome) in SoyBase (http://www.soybase.org/). We carried out Gene Ontology (GO) enrichment analysis for all the genes within the three major QTL intervals using OmicShare Tools (http://www.omicshare.com/tools). The Guizao1 and B13 resequencing data were used to identify candidate genes possessing nonsynonymous SNV or InDel mutations in coding regions. The available RNA-Seq dataset from SoyBase (http://www.soybase.org/soyseq/) was downloaded and used to analyze the expression of candidate genes in different developmental stages of soybean tissues [62]. In summary, these methods were used in combination to identify credible candidate genes.

## Supplementary Information

**Additional file 1: Table S1.** The significant LOD thresholds were determined by 1000permutations (*p* < 0.05). **Table S2.** Overall genes related toseed Fe and Zn content in three major QTL regions. **Table S3.** GeneOntology (GO) enrichment analysis of overall genes in three major QTL regions. **TableS4.** Significant SNV mutations and mutant genes between Guizao1 and B13 inthree major QTLs. **Table S5.** Significant InDel mutations and mutant genesbetween Guizao1 and B13 in three major QTLs. **Table S6.** Expression analysisof mutant genes in soybean in different developmental stages and tissues. **Table S7.** Variations inputative candidate genes within *qFC7*, *qZC3*, and *qZC20*. **Table S8.**Raw phenotyping data of GB RIL population and two parents collected in four environments.

**Additional file 2: Fig. S1.** The significant differences between Guizao1 and B13 for seed Fe andZn content Histogramsshowing the significant differences between Guizao1 and B13 for seed Fe and Zn content in thefour environments, according to the ANOVA analysis denoted asfollows: **p < 0.01. **Fig. S2.** The expression patterns analysis of mutant genes from three major QTLs Heatmap showing the expression patternsof mutant genes from three major QTLsamong the different tissues during soybean development stages based on publicRNA-seq data from SoyBase. DAF: days after flowering. a, b, and c representmutant genes from *qZC3*, *qFC7*, and *qZC20, *respectively.

## Data Availability

The data that support the findings of this study are available from the Genome Sequence Archive database at the National Genomics Data Center, Beijing Institute of Genomics (BIG), Chinese Academy of Sciences, with accession number CRA004753 (https://bigd.big.ac.cn/gsa/browse/CRA004753) and CRA004754 (https://bigd.big.ac.cn/gsa/browse/CRA004754). The phenotype dataset used during the current study is provided in the Addational file 1: Table S8.

## Abbreviations

Fe: Iron
Zn: Zinc
QTL: Quantitative trait loci
RIL: Recombinant inbred line
MAS: Marker-assisted selection
NGS: Next-generation sequencing
SD: Standard deviation
CV: Coefficient of variance
*H*^*2*^: Broad-sense heritability
Chr: Chromosome
CIM: Composite interval mapping method
ICIM-ADD: Inclusive composite interval mapping of the additive method
QEI: QTL × environment interaction
LOD: Logarithm of odds
RNA-Seq: RNA sequencing
GO: Gene Ontology
SNP: Single nucleotide polymorphism
SNV: Single-nucleotide variants
InDel: Insertion and deletion
