# Supplementary figures and images for: Identification of quantitative trait loci (QTLs) and candidate genes of seed Iron and zinc content in soybean [Glycine max (L.) Merr.]
Source: BMC Genomics. 2022 Feb 19;23:146. doi: 10.1186/s12864-022-08313-1 (PMC8857819; doi:10.1186/s12864-022-08313-1)

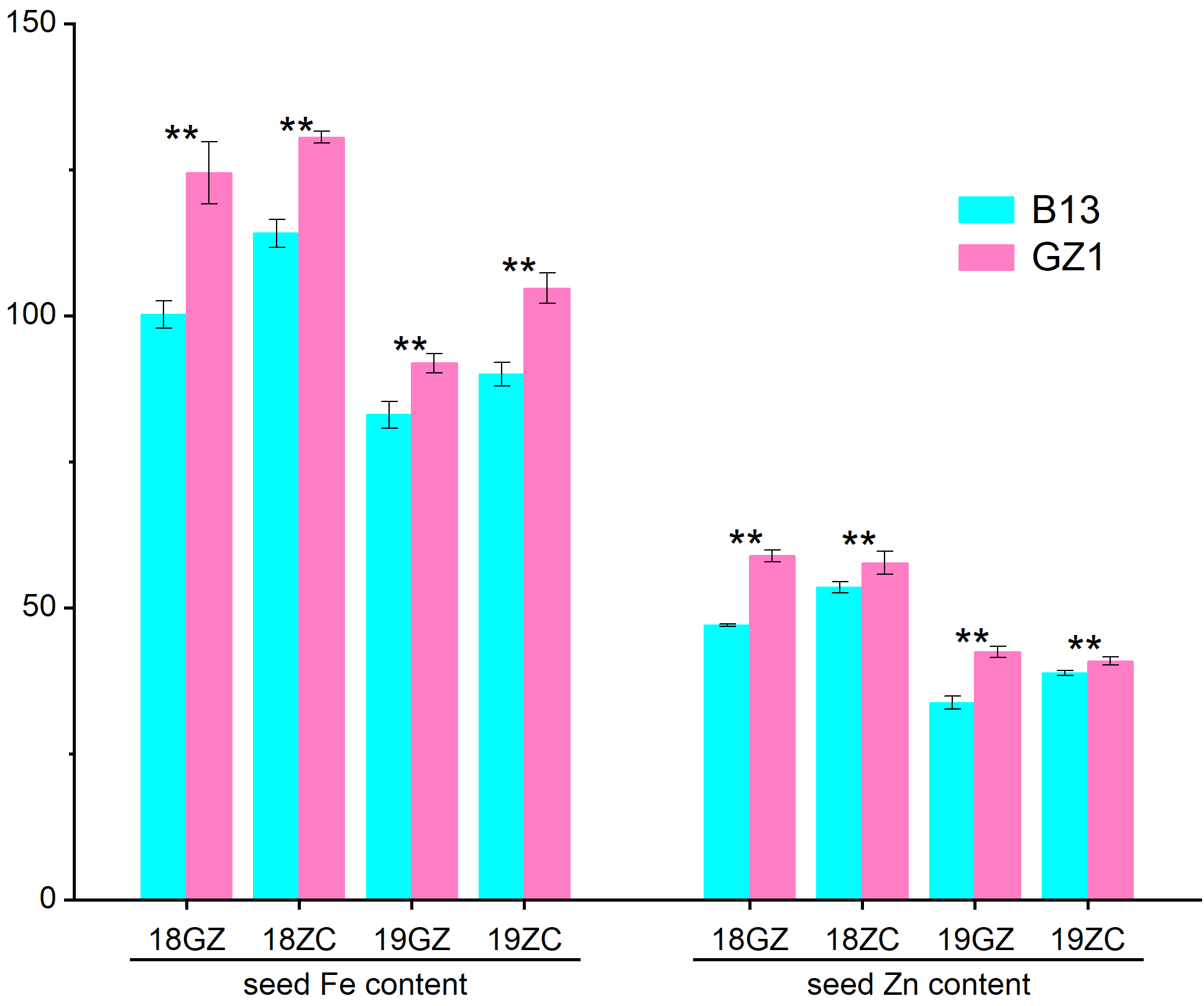

Supplement: Supplementary file 2 — Additional file 2: Fig. S1. The significant differences between Guizao1 and B13 for seed Fe andZn content Histogramsshowing the significant differences between Guizao1 and B13 for seed Fe and Zn content in thefour environments, according to the ANOVA analysis denoted asfollows: **p < 0.01. Fig. S2. The expression patterns analysis of mutant genes from three major QTLs Heatmap showing the expression patternsof mutant genes from three major QTLsamong the different tissues during soybean development stages based on publicRNA-seq data from SoyBase. DAF: days after flowering. a, b, and c representmutant genes from qZC3, qFC7, and qZC20, respectively. [file 12864_2022_8313_MOESM2_ESM.zip › Additional file 2/Fig. S1.tif]
